# Association of genetic and climatic variability in giant sequoia, *Sequoiadendron giganteum*, reveals signatures of local adaptation along moisture‐related gradients

**DOI:** 10.1002/ece3.6716

**Published:** 2020-09-01

**Authors:** Rainbow DeSilva, Richard S. Dodd

**Affiliations:** ^1^ Department of Environmental Science, Policy, and Management University of California at Berkeley Berkeley California USA

**Keywords:** climate change, genotype by sequencing, giant sequoia, landscape genomics, local adaptation

## Abstract

Uncovering the genetic basis of local adaptation is a major goal of evolutionary biology and conservation science alike. In an era of climate change, an understanding of how environmental factors shape adaptive diversity is crucial to predicting species response and directing management. Here, we investigate patterns of genomic variation in giant sequoia, an iconic and ecologically important tree species, using 1,364 bi‐allelic single nucleotide polymorphisms (SNPs). We use an *F*_ST_ outlier test and two genotype–environment association methods, latent factor mixed models (LFMMs) and redundancy analysis (RDA), to detect complex signatures of local adaptation. Results indicate 79 genomic regions of potential adaptive importance, with limited overlap between the detection methods. Of the 58 loci detected by LFMM, 51 showed strong correlations to a precipitation‐driven composite variable and seven to a temperature‐related variable. RDA revealed 24 outlier loci with association to climate variables, all of which showed strongest relationship to summer precipitation. Nine candidate loci were indicated by two methods. After correcting for geographic distance, RDA models using climate predictors accounted for 49% of the explained variance and showed significant correlations between SNPs and climatic factors. Here, we present evidence of local adaptation in giant sequoia along gradients of precipitation and provide a first step toward identifying genomic regions of adaptive significance. The results of this study will provide information to guide management strategies that seek to maximize adaptive potential in the face of climate change.

## Graphical abstract

We investigate patterns of genomic variation in giant sequoia, an iconic and ecologically important tree species, using 1,364 bi‐allelic single nucleotide polymorphisms (SNPs). Using genotype–environment association and *F*_ST_ outlier tests, we identify 79 genomic regions of potential adaptive importance, with limited overlap between the detection methods. The majority of the outlier loci show strong correlations to moisture‐related variables indicating local adaptation of giant sequoia populations along gradients of precipitation.

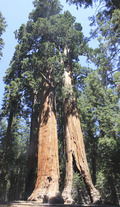

## 1 INTRODUCTION

In an era of unprecedented climate change, the adaptive potential of populations has become an increasingly important topic to conservation biologists, raising questions of landscape partitioning of adaptive variation and management strategies to maintain population viability. Given the rapid rate of climate change, new beneficial mutations are expected to play a limited role for species with low mutation rates and long generation times. Therefore, adaptive evolution under climate change for many species will depend on standing genetic variation (Aitken, Yeaman, Holliday, Wang, & Curtis‐McLane, 2008; Barrett & Schluter, 2008) that may vary across the landscape and include alleles that gain adaptive value as selection pressures change (Olson‐Manning, Wagner, & Mitchell‐Olds, 2012). Reliance on standing genetic variation is likely to be particularly true for long‐lived sedentary species, such as forest trees that are characterized by adaptive constraints that can limit their evolutionary response to rapid environmental change: extended generation times that result in local persistence, increased rates of genetic drift associated with overlapping generations (Rogers & Prügel‐Bennett, 2000), and limited rates of migration due to long generation times. For these species, understanding the distribution of adaptive diversity in relation to recent or past climatic gradients is a critical first step in promoting the adaptive potential of populations in hopes of maintaining future viability (Aitken & Whitlock, 2013; Holderegger, Kamm, & Gugerli, 2006).

The rich history of field research on phenotypic traits in plants (common gardens and reciprocal transplant studies) provides evidence for abundant heritable variation for quantitative traits that are organized along environmental clines (Morgenstern, 1996; Savolainen, Pyhäjärvi, & Knurr, 2007). Until recently, determining the molecular basis of this variation has been less tractable. However, the rapid advancement of genome sequencing, including methods that use reduced genomic complexity (e.g., genotyping by sequencing (GBS), restriction‐site associated DNA sequencing (RADseq)), has opened the door to more comprehensive assessments of population‐level diversity and allowed for the detection of regions under selection. Although some instances of strong selection on single or few gene loci have been noted (Akey, 2009; Linnen, Kingsley, Jensen, & Hoekstra, 2009; Sella, Petrov, Przeworski, & Andolfatto, 2009), many traits of adaptive importance in plants are believed to be polygenic in nature (Holland, 2007; Le Corre & Kremer, 2012; Pritchard & Di Rienzo, 2010; Yeaman et al., 2016). Under selection, these traits can exhibit subtle changes in frequency across many loci of small effect. Further, demographic processes can shape genetic diversity in ways that mimic selective gradients, as geographic distance and climatic gradients are often autocorrelated. As a result, imprints of selection within the genome can be difficult to detect (Yeaman, 2015), and it is necessary to parcel out the contribution of geographic space in order to successfully identify regions of functional importance (Excoffier, Hofer, & Foll, 2009; Rellstab, Gugerli, Eckert, Hancock, & Holderegger, 2015).

By coupling genome‐wide markers with landscape genomics analyses, many researchers have successfully uncovered patterns of adaptive variation and identified potential genomic regions under selection across a wide variety of species (Benestan et al., 2016; De Kort et al., 2014; Dudaniec, Yong, Lancaster, Svensson, & Hansson, 2018; Harrisson et al., 2017; Lind et al., 2017; Pais, Whetten, & Xiang, 2016). *F*_ST_ outlier tests, that scan for highly differentiated loci as candidates for divergent selection, have proven useful in detecting regions under selection but often cannot detect weak or polygenic selection (Lotterhos & Whitlock, 2015; Narum & Hess, 2011; Pritchard & Di Rienzo, 2010). Genotype–environment association (GEA) tests have demonstrated high power to detect signals of adaptive evolution under varying demographic scenarios (Forester, Lasky, Wagner, & Urban, 2018; Lotterhos & Whitlock, 2015; de Villemereuil, Frichot, Bazin, François, & Gaggiotti, 2014). Univariate association methods that test for single‐locus–single‐predictor correlation after accounting for population structure are powerful tools to accurately detect even weak signatures of adaptation (Frichot, Schoville, Bouchard, & François, 2013; Gunther & Coop, 2013; Lotterhos & Whitlock, 2015; Rellstab et al., 2015; de Villemereuil et al., 2014). However, a shortcoming of assessing each locus independently is a potential failure to detect signals of polygenic selection (Forester et al., 2018). Multivariate approaches can fill this gap by assessing the combined effects of multiple loci and predictors (Capblancq, Luu, Blum, & Bazin, 2018; Forester et al., 2018; Rellstab et al., 2015), which is perhaps more reflective of real‐life evolutionary pressures. Given the advantages of each method, combining outlier tests with GEA can increase the likelihood of detecting complex patterns of selection (Rellstab et al., 2015).

Determining the presence of adaptively important genetic variation and its distribution across a species range is crucial to predicting species' responses to global climate change and directing biodiversity conservation and management efforts (Aitken & Whitlock, 2013; Alberto et al., 2013; Funk, McKay, Hohenlohe, & Allendorf, 2012; Sgrò, Lowe, & Hoffmann, 2011; Sork et al., 2013). This has become an urgent challenge in California, where a protracted drought has resulted in massive tree mortality (USDA, 2016). The Sierra Nevada of California is a high mountain range that collects precipitation from the Pacific Ocean mostly in the form of winter rain and snowfall. The slow release of water from snowmelt in the spring is an important source of moisture for seedling growth and establishment. Sierra snowpack has declined in recent years (Fyfe et al., 2017) and high‐resolution regional climate models suggest that spring snow water equivalent will decline by 73% by the end of the century, with midelevations (1,500–2,500 m) experiencing the greatest declines (Sun, Berg, Hall, Schwartz, & Walton, 2018).

**FIGURE 1:**
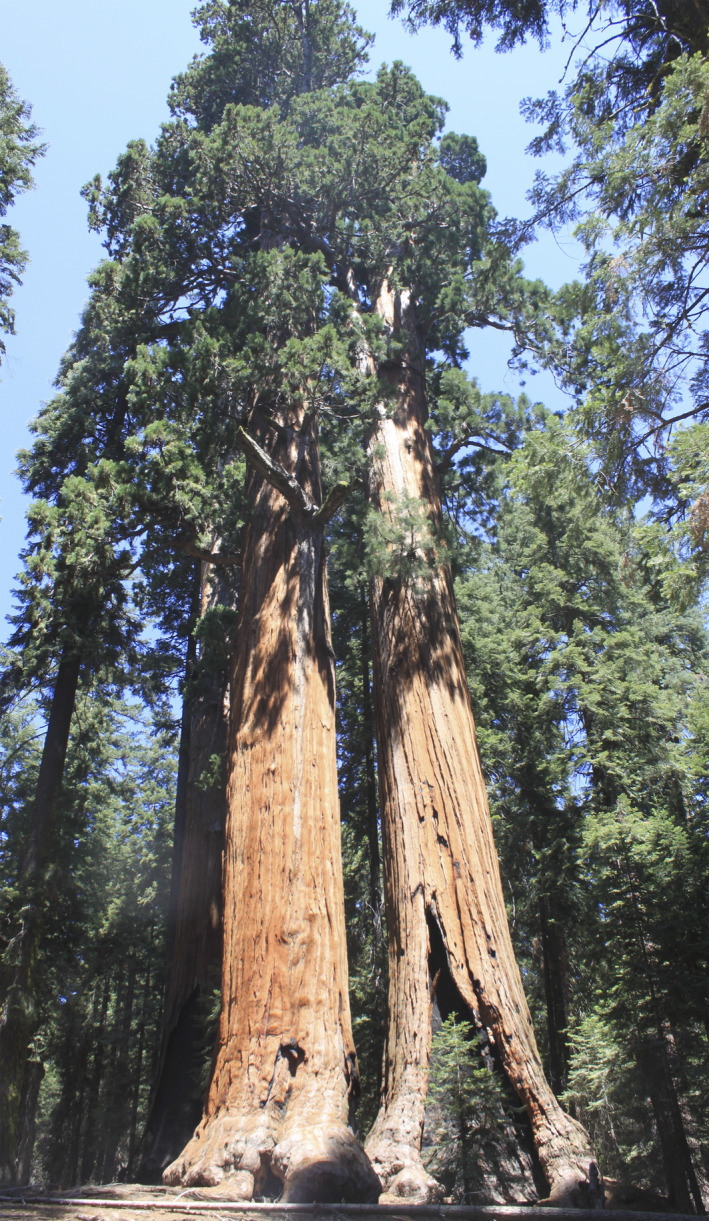
A giant sequoia tree in Giant Forest, Sequoia and Kings Canyon National Park, CA, USA

**FIGURE 2:**
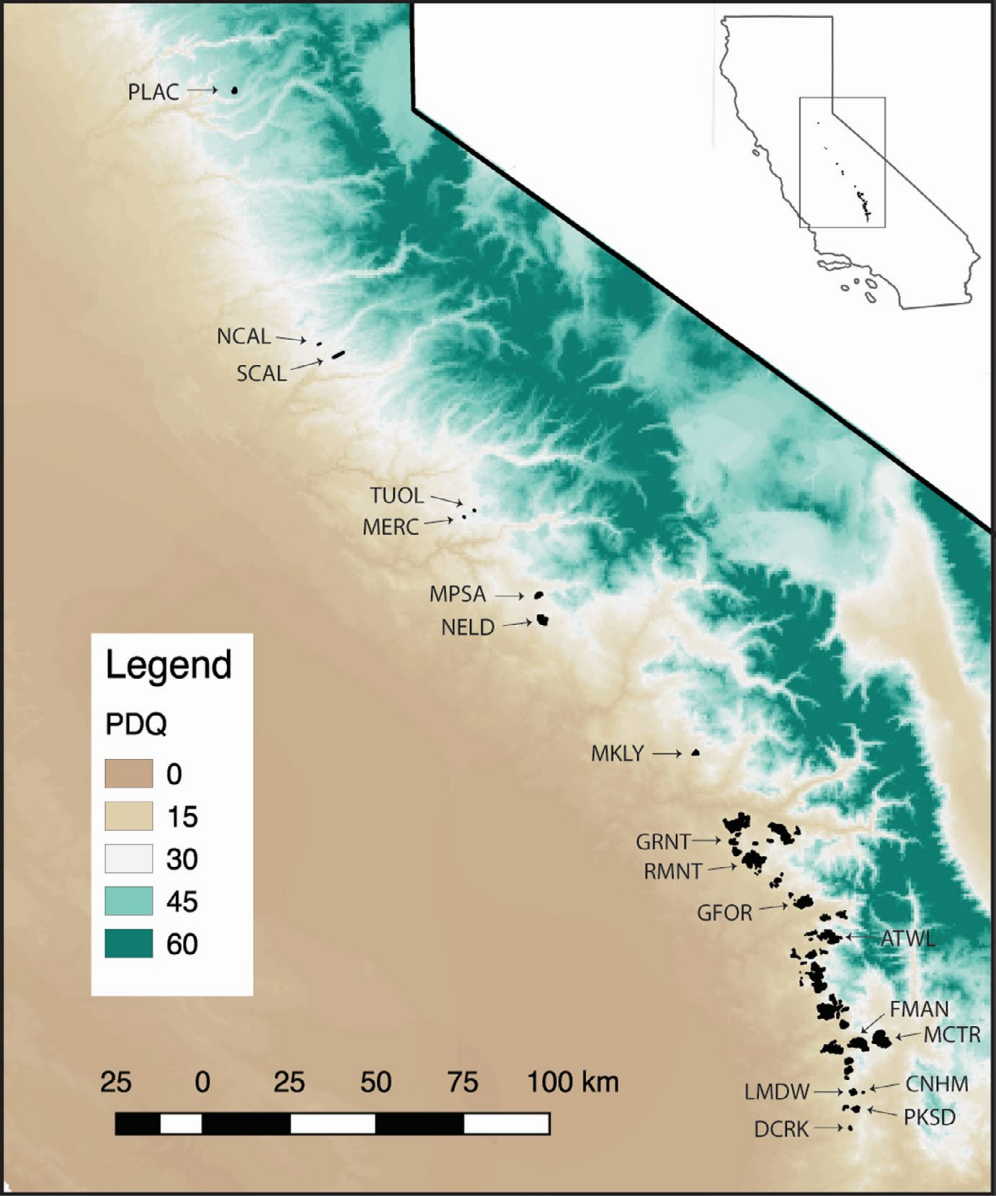
Range map of giant sequoia (black) showing the gradient of precipitation of driest quarter (mm) across a section of California. Sampled populations indicated by a population code

This elevational range includes the extant groves of the iconic, long‐lived conifer, giant sequoia (*Sequoiadendon giganteum* [Lindl.] Buchholz) that occur in a highly disjunct range consisting of ~70 groves spanning approximately 400 km north to south (Figures 1 and 2). Currently, most giant sequoia populations are in protected areas as this species is valued both culturally and for ecotourism. However, despite this protected status, a key question is whether populations of giant sequoia will remain viable under changing climate. Our previous work has shown very restricted gene flow (DeSilva & Dodd, 2020), suggesting that natural dispersal outside of existing groves will be unlikely. Long generation times (~305 years; Dodd & DeSilva, 2016) will slow the expansion of new variants that may arise through mutation, which underscores the role of standing genetic variation in determining the future viability of giant sequoia populations. In our landscape genetics study of microsatellite variation, we found some evidence for isolation by environment (IBE), linking genetic divergence at putatively neutral loci to dissimilarity in precipitation, and temperature‐related variables (DeSilva & Dodd, 2020). Although IBE is consistent with local adaptation, it is dependent on a reduction of gene flow from divergent habitats due to selection against nonadapted immigrants, and therefore, patterns of IBE may not be reflective of local adaptation when gene flow is low or absent (Nosil, Vines, & Funk, 2005; Wang & Bradburd, 2014), as is likely the case in sections of giant sequoia range (DeSilva & Dodd, 2020). A recent common garden study reported provenance variation in growth performance, providing support for the existence of adaptive genetic variation across the species' range (Valness, 2016). Yet, to date, no studies have investigated local adaptation in giant sequoia using genomic data. Our ultimate goal was to detect populations that may be genetically responsive to anticipated climate change, so here, we build upon this earlier work by reporting on genomic signatures of selection using a range‐wide genotyping‐by‐sequencing dataset. Specifically, we utilize an *F*_ST_ outlier test and gene–environment association methods (LFMM and RDA), to find signatures of local adaptation among giant sequoia populations and locate potential genomic regions under selection.

## 2 METHODS

### 2.1 DNA extraction, GBS library preparation, and data processing

Foliage was collected from 6 to 9 trees within each of 18 populations of giant sequoia distributed throughout the range (Figure 2). To reduce the potential of sampling related individuals, we aimed to sample individual trees >40 m apart. However, this was not possible in some small and highly clustered populations. In this latter case, we attempted to maximize the distance between sampled individuals, with the exception of the PLAC population, where all individuals were sampled. Our goal was to maximize the capture of variation across the range of our study species. Thus, we prioritize increased sampling of populations across the *S. giganteum* range, with the trade‐off of limited sampling within each population. Appropriate permits were obtained for all sampling.

High‐purity genomic DNA from 143 individuals was isolated from leaf tissue using Plant/Fungi DNA Isolation kits (Norgen Biotek). We constructed three sequencing libraries using a double‐digest restriction enzyme‐associated genotyping‐by‐sequencing (GBS) protocol outlined in Peterson, Dong, Horback, and Yong‐Bi (2014). Genomic DNA was digested using SbfI and EcoRI restriction enzymes (New England Biolabs). The resulting product was ligated to barcoded adapters and purified, and 46–48 individuals per library were then pooled and subjected to PCR amplification using Phusion High‐Fidelity PCR Kit (New England Biolabs) and an automated size selection for fragments between 430 and 570 bp using Pippen Prep. The resulting three libraries were sequenced on an Illumina HiSeq 4000 platform using 150 bp pair‐end reads. Sequence data were then demultiplexed using the process_radtags module within the STACKS pipeline (Catchen, Hohenlohe, Bassham, Amores, & Cresko, 2013), during which reads with a phred quality score < 10 were removed. Sequences were then aligned to the giant sequoia reference genome v1.0 (Redwood Genome Project, 2019), using the software Bowtie 2 and SAMtools (Langmead & Salzberg, 2012; Li et al., 2009). Variable sites were called using FreeBayes (Garrison & Marth, 2012) and filtered to remove low‐quality reads, potential sequencing errors, and paralogs. Data filtering steps included removing loci with uneven mapping quality and those with average read depth >200, requiring a minimum read depth of 5× and a minor allele count >3, removal of loci with more than 80% missing data, and a thinning step that retains one SNP per DNA fragment to remove potentially linked loci (Appendix S1). This filtering protocol resulted in a final dataset of 1,364 bi‐allelic SNPs used for outlier tests and environmental association analyses and to obtain genetic diversity statistics.

### 2.2 Environmental data

To characterize the climatic conditions for each population, we used the spatial centroid of each population to extract and compile twenty‐one environmental variables at a spatial resolution of approximately 1 km^2^. Nineteen climate variables were obtained from the WorldClim database (Fick & Hijmans, 2017), and elevation and climate water deficit (CWD) were obtained from the California Basin Characterization Model (Flint, Flint, Thorne, & Boynton, 2013). CWD provides an indication of aridity that is important for Mediterranean climate systems, such as in California (Stephenson, 1998). We conducted a principal component analysis (PCA) on the full environmental dataset (21 variables) after standardization, to reduce dimensionality in the climate data. We retained the first two axes (hereafter PC1 and PC2), which together explained 82% of the climate variation (Appendices S2 and S3). PC1 was driven predominantly by temperature and elevation variables, with a small contribution from annual and winter precipitation, whereas PC2 was determined mostly by precipitation‐related variables and CWD with a minor contribution from variables related to temperature seasonality (Appendices S2 and S3).

### 2.3 Genetic diversity

Genetic diversity and differentiation statistics were calculated using both the “diveRsity” package in R and GenoDive (Keenan, McGinnity, Cross, Crozier, & Prodöhl, 2013; Meirmans & Van Tienderen, 2004). Calculated statistics included observed and unbiased expected heterozygosity (H_o_ and uHe, respectively), the inbreeding coefficient (*F*_IS_), and the pairwise fixation index (*G*′_ST_). Since the removal of rare alleles (minor allele count filtering) can bias genetic diversity estimation, we also calculated genetic diversity statistics without this filtering step for comparison. To further investigate the partitioning of genetic variation, we used AMOVA with 10,000 permutations to estimate *F*_ST_ across all populations as well as between northern and southern regions which previous evidence suggested were divergent (DeSilva & Dodd, 2020; Dodd & DeSilva, 2016). For regional diversity comparisons, groves north of GRNT were grouped as northern populations and groves from GRNT to the south as southern populations (Figure 2; DeSilva & Dodd, 2020).

### 2.4 Genomic signatures of selection: *F*_ST_ outliers and gene–environment association tests

To detect *F*_ST_ outliers that are candidates for selection, we utilized the Bayesian likelihood approach implemented in BayeScan v.2.1 (Foll & Gaggiotti, 2008). This method scans the genome for highly differentiated SNPs that potentially have been subjected to divergent selection while accounting for neutral genetic structure (Narum & Hess, 2011). BayeScan was run using the false discovery rate (FDR) set to 0.05 under the following parameters: 20 pilot runs of 5,000 with an additional burn in of 50,000 iterations and a subsequent run with 5,000 iterations and a thinning interval of 10. The prior odds for the neutral model were increased to 100 (default is 10) as raising this value has been shown to reduce false positives with little effect on false negatives (Lotterhos & Whitlock, 2014). Loci with log_10_ values of the posterior odds >1.0 were retained, as the program documentation suggests these loci show “strong” evidence for selection (Foll, 2010).

To test for associations between genomic variation and environmental factors, we utilized latent factor mixed models (LFMMs; Frichot et al., 2013), as implemented in the LEA package in R (Frichot & François, 2015a). LFMM is a univariate approach that treats each individual locus as a response variable with climate data (PC1 and PC2 separately) as the explanatory variable, while incorporating neutral structure using latent factors (Frichot et al., 2013). In simulation studies, LFMM has demonstrated a good balance between high power and low false‐positive rate (Frichot et al., 2013; de Villemereuil et al., 2014). As suggested by Frichot et al. (2013) and Frichot and François (2015a), we used two methods to determine the optimal number of latent factors (*K* value) that correct for the neutral genetic structure of our data. First, we ran a principal component analysis on the individual allele frequencies. We then determined the number of components that explain the genetic variance, based on the Tracy–Widom test on the eigenvalues, as an estimate of K (Frichot & François, 2015a). Second, we utilized the Bayesian clustering algorithm STRUCTURE that estimates the number of genetic clusters (*K*) without prior information about geographic origin (Pritchard, Stephens, & Donnelly, 2000). The best *K* value was determined using the ∆*K* statistic as suggested by Evanno, Regnaut, and Goudet (2005). We used four replicates and a burnin of 300,000 and 1,000,000 MCMC repeats after burnin for *K* = 2–12.

We ran LFMM to test for associations between SNP′s and two composite climate variables (PC1 and PC2) using ten independent replications at 50,000 iterations after a burnin period of 25,000 with the number of latent factors (K) ranging from 8 to 12, as the methods outlined above suggested *K* equal to 10 and 9, respectively. We chose high run length parameters because of the relatively small number of individuals and loci. LFMM uses the *z*‐scores to indicate the strength of the gene–environment association (Frichot & François, 2015b). As suggested by the authors, we calculated the median *z*‐score from ten replicate runs, re‐adjusted the p‐values, controlled for FDR using the *q*‐value of 0.05, and determined candidate SNPs based on the Benjamini–Hochberg procedure (Frichot & François, 2015b).

We also utilized RDA, a multivariate GEA method, to test for more subtle polygenic signatures of adaptation and detect outlier loci as candidates of functional importance. Redundancy analysis (RDA) is an extension of multiple regression to multivariate response variables (Legendre & Legendre, 2012). In finding the ideal combination of predictor and explanatory variables, RDA has shown high power to detect potential signals of polygenic adaptation (Forester et al., 2018; Harrisson et al., 2017). For these analyses, Hellinger‐transformed allele frequencies (Legendre & Gallagher, 2001) were treated as response variables. Because RDA models do not allow missing data, we imputed allele frequency data using probabilistic principal component analysis (ppca) as implemented in the “pcaMethods” package in R (Stacklies, Redestig, Scholz, Walther, & Selbig, 2007). Ppca uses a decomposition of SNP frequencies to create principal components; the components with the largest eigenvalues are then used to impute the missing data. We evaluated space and climate as explanatory variables. Space was defined by distance‐based Moran's eigenvector maps (dbMEMs; Borcard & Legendre, 2002; Dray, Legendre, & Peres‐Neto, 2006) based on Euclidean distances between all giant sequoia groves (sampled and unsampled) and extracting the values that correspond to our sample sites. Then, we conducted backward model selection, using the “ordistep” function within the vegan package for R (Oksanen et al., 2013), to reduce the number of dbMEM vectors. For climate, we reduced the twenty‐one untransformed environmental variables described above, first by removing highly correlated environmental variables, (|*r*| < 0.7), and subsequently by using the “ordistep” function for backwards model selection to remove variables lacking explanatory power. The above process resulted in climate being represented by “isothermality” (ISO), a measure diurnal and annual temperature fluctuation, “precipitation of driest quarter” (PDQ), a measure of summer precipitation in Mediterranean climates, and “climate water deficit” (CWD), a measure of aridity, in all RDA models, and space represented by two dbMEM vectors, MEM3 and MEM5. All variables were centered and standardized before use in each model.

We set up multiple RDA models to determine the relative amount of variation in allele frequency explained by climate after correcting for geographic space as a signature of local adaptation (Harrisson et al., 2017; Lasky et al., 2012; Sork et al., 2016). First, to elucidate the major factors shaping genetic variation and to detect potential signals of local adaptation, we set up three models for comparison: a full RDA model where allele frequencies were associated with both climate and spatial explanatory variables, a partial RDA in which the effects of climate were conditioned on geography (dbMEMs), and a second partial RDA, where the effects of geography were conditioned on climate. Next, to detect outlier loci, allele frequencies were associated with climate predictors after removing the effects of spatial predictors (Forester et al., 2018; Harrisson et al., 2017; Lasky et al., 2012). Using the first constrained axis, we identified candidate SNPs of potential adaptive importance as those with loadings in the tails of a 95% confidence interval from the mean or 2.0SD from the mean loadings. One risk of using such a low cutoff is an elevated rate of false positives. However, we chose this to maximize the number of SNPs detected, as we did not expect to find single loci that would be under very strong selection for climate variation in the range of giant sequoia. Moreover, we also identified the climate predictor with the highest correlation to each indicated SNP. In all RDA models, we assessed model and constrained‐axis significance using 999 permutations.

### 2.5 Genomic context of outlier loci

To gain insights into the potential adaptive significance of outlier loci, we obtained the flanking sequence of each outlier SNP locus from the giant sequoia reference sequence (Redwood Genome Project, 2019). Since the giant sequoia reference genome is not annotated, functional annotation was performed using the online BLAST (Basic Local Alignment Search Tool) database. Using a 601‐bp sequence (300 bp upstream and downstream of the SNP site), we searched the NCBI database using BLASTn with an *e*‐value cutoff set to 1 × 10^5^ and the requirement of >70% sequence similarity.

## 3 RESULTS

### 3.1 Genetic diversity and differentiation

Genetic diversity and differentiation differed substantially across the eighteen sampled populations (Table 1). Observed heterozygosity (Ho) ranged from 0.09 to 0.17 and was lowest in PLAC and highest in ATWL and FMAN (Table 1). Unbiased expected heterozygosity (uHe) was also lowest in PLAC (0.07) and highest in ATWL (0.21) (Table 1). Average pairwise population differentiation (*G*′_ST_) varied from 0.09 to 0.32 and was lowest for GFOR and highest for PLAC (Table 1). Average *G*′_ST_ was significantly higher in the northern populations than in the southern populations (*G*′_ST_ N = 0.235, *G*′_ST_ S = 0.109, Prob *G*′_ST_ N ≠ *G*′_ST_ S = 0.017). Diversity analysis of SNPs without minor allele count (MAC) filtering yielded significantly different results: Ho and uHe were lower and ranged from 0.08 to 0.15 and 0.06 to 0.18, respectively (*p* < .001, *p* = .01 respectively, Table 1), whereas *G*′_ST_ was slightly higher in the dataset without MAC filtering (*G*′_ST_ 0.09–0.34, *p* = .003, Table 1).

**TABLE 1:** Population information and genetic diversity summary statistics calculated for each population. Diversity statistics calculated without minor allele filtering are noted within parentheses.

Grove name	Population code	GPS location	Sample size	Ho	uHe	*F*_IS_	*Mean G*′st
Placer	PLAC	39.06, −120.57	6	0.09 (0.08)	0.07 (0.06)	0.00 (0.01)	0.32 (0.34)
North Calaveras	CALN	38.28, −120.30	8	0.14 (0.13)	0.19 (0.16)	0.17 (0.17)	0.17 (0.17)
South Calaveras	CALS	38.24, −120.25	8	0.16 (0.14)	0.18 (0.16)	0.13 (0.13)	0.15 (0.15)
Tuolumne	TUOL	37.77, −119.81	8	0.16 (0.14)	0.17 (0.14)	−0.04 (−0.04)	0.24 (0.25)
Merced	MERC	37.75, −119.84	8	0.16 (0.14)	0.17 (0.15)	0.00 (−0.01)	0.21 (0.21)
Mariposa	MPSA	37.51, −119.60	8	0.13 (0.11)	0.14 (0.12)	0.11 (0.11)	0.20 (0.20)
Nelder	NELD	37.43, −119.59	8	0.16 (0.15)	0.20 (0.17)	0.13 (0.11)	0.13 (0.14)
McKinley	MKLY	37.03, −119.11	8	0.15 (0.13)	0.17 (0.15)	0.07 (0.06)	0.17 (0.17)
Grant	GRNT	36.75, −118.97	8	0.12 (0.10)	0.13 (0.11)	0.17 (0.18)	0.14 (0.15)
Redwood Mountain	RMNT	36.60, −118.92	8	0.12 (0.10)	0.11 (0.10)	0.17 (0.18)	0.13 (0.13)
Giant Forest	GFOR	36.57, −118.76	8	0.15 (0.14)	0.20 (0.18)	0.18 (0.17)	0.09 (0.09)
Atwell	ATWL	36.47, −118.67	8	0.17 (0.15)	0.21 (0.18)	0.08 (0.08)	0.11 (0.12)
Mcintyre	MCTR	36.13, −118.58	8	0.13 (0.11)	0.11 (0.09)	0.10 (0.11)	0.12 (0.13)
Freeman Creek	FMAN	36.14, −118.52	8	0.17 (0.12)	0.19 (0.16)	0.08 (0.07)	0.10 (0.11)
Long Meadow	LMDW	35.96, −118.60	8	0.12 (0.11)	0.11 (0.10)	0.16 (0.15)	0.15 (0.16)
Cunningham	CNHM	35.92, −118.57	9	0.16 (0.15)	0.17 (0.16)	0.00 (0.00)	0.16 (0.16)
Packsaddle	PKSD	35.93, −118.59	8	0.15 (0.13)	0.15 (0.13)	0.08 (0.08)	0.16 (0.16)
Deer Creek	DCRK	35.88, −118.61	8	0.16 (0.14)	0.18 (0.16)	0.05 (0.04)	0.16 (0.16)

**FIGURE 3:**
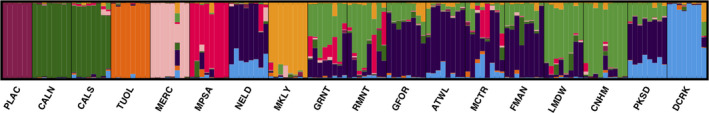
Results of population structure analyses from STRUCTURE. Vertical bars represent a sampled individual, color‐coded for assigned cluster at *K* = 9

Hierarchical AMOVA found a small, but significant variance due to regions (*F*ct = 0.02, *p* = .000) and a larger portion of genomic variation distributed among populations (*F*_ST_ = 0.15, *p* = .000; Appendix S4). Population clustering (STRUCTURE) at *K* = 9 indicated strong differentiation among many of the northern populations (north of GRNT) with little admixture (Figure 3, Appendix S5). In addition, populations NELD, GFOR, ATWL, GRNT, MCTR, and FMAN were assigned to the same cluster and the four southernmost populations, LMDW, CNHM, PKSD, and DCRK, consisted of two clusters with PKSD as a transitional population exhibiting admixture from both clusters (Figure 3). Finally, RMNT and GRNT show admixture between the neighboring GFOR, ATWL, GRNT, MCTR, FMAN, cluster, and the geographically separate LMDW, CNHM, cluster (Figure 3).

### 3.2 *F*_ST_ outliers

BayeScan indicated seven *F*_ST_ outliers, six demonstrating evidence for divergent selection with *F*_ST_ values ranging from 0.55 to 0.72 (Table 2, Appendix S6) and one showing signs of balancing selection (locus 1,114, *F*_ST_ = 0.04). Since our focus here is on patterns of spatially varying selection, no further discussion is presented for locus 1,114. A BLAST search found one of these loci exhibited functional significance (Locus 828; Table 2).

**TABLE 2:** Functional significance, detection method, and associated variable for highly supported outlier loci

Locus ID	Detection method	Associated variable* (*F*_ST_)	Adjusted *p*‐value	| Axis 1 Loading |	Annotation
828	BayeScan	(0.58)			Unknown mRNA
1,123	BS, LFMM	PC1(0.66)	1.72E‐04		—
186	LFMM	PC1	7.39E‐06	—	Unknown mRNA
218	LFMM	PC1	5.50E‐05	—	Unknown mRNA
452	LFMM	PC1	5.30E‐05	—	Unknown mRNA
251	LFMM	PC2	1.91E‐05	—	Unknown mRNA
722	LFMM	PC2	1.52E‐03	—	Unknown mRNA
870	LFMM	PC2	9.48E‐04	—	Unknown mRNA
1,029	LFMM	PC2	6.56E‐06	—	Unknown mRNA
1,062	LFMM	PC2	1.66E‐04	—	Unknown mRNA
1,214	LFMM	PC2	1.52E‐03	—	Magnesium transporter MRS2‐4‐like
1,253	LFMM	PC2	2.91E‐04	—	Unknown mRNA
1,313	LFMM	PC2	5.87E‐06	—	Pleiotropic drug resistance protein 1‐like
368	LFMM, RDA	PC2, PDQ	2.35E‐05	0.21132	—
421	LFMM, RDA	PC2, PDQ	9.56E‐05	0.19423	—
471	LFMM, RDA	PC2, PDQ	2.03E‐05	0.19892	—
515	LFMM, RDA	PC2, PDQ	1.46E‐03	0.21181	—
679	LFMM, RDA	PC2, PDQ	3.51E‐04	0.22348	—
827	LFMM, RDA	PC2, PDQ	9.12E‐04	0.20579	Kinesin‐like protein KIN‐13A mRNA
1,229	LFMM, RDA	PC2, PDQ	1.65E‐03	0.17822	Pollen allergen gene
1,286	LFMM, RDA	PC2, PDQ	4.41E‐07	0.26369	—
338	RDA	PDQ	—	0.18769	Wall‐associated receptor kinase‐like 1
612	RDA	PDQ	—	0.24267	Arogenate dehydratase gene
617	RDA	PDQ	—	0.17057	Unknown mRNA
940	RDA	PDQ	—	0.23536	Signal peptidase I AT2G30440 mRNA
1,066	RDA	PDQ	—	0.19520	Unknown mRNA

### 3.3 Candidate genomic regions associated with climate variables

**FIGURE 4:**
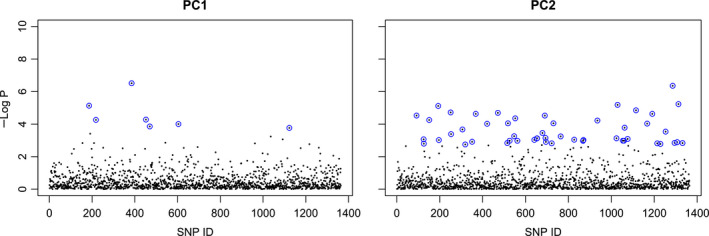
Adjusted *p*‐values from LFMM for association with PC1 and PC2. Outliers are outlined in blue

Univariate environmental association analyses (LFMM with *K* = 9) indicated a total of 58 loci with strong correlations to composite environmental variables (Figure 4; Appendix S6). Of these, 51 were correlated with PC2 that was predominantly driven by precipitation, and seven were correlated with the temperature‐driven PC1 (Figure 4; Table 2; Appendix S6). An examination of the adjusted P‐values from all runs (*K* set from 8–12) provided additional support for *K* = 9 (Appendix S7; Frichot & François, 2015b). Our BLAST analysis was successful in finding functional annotation for three loci that were strongly associated with PC1 and 11 loci that were associated with PC2 (Table 2).

**FIGURE 5:**
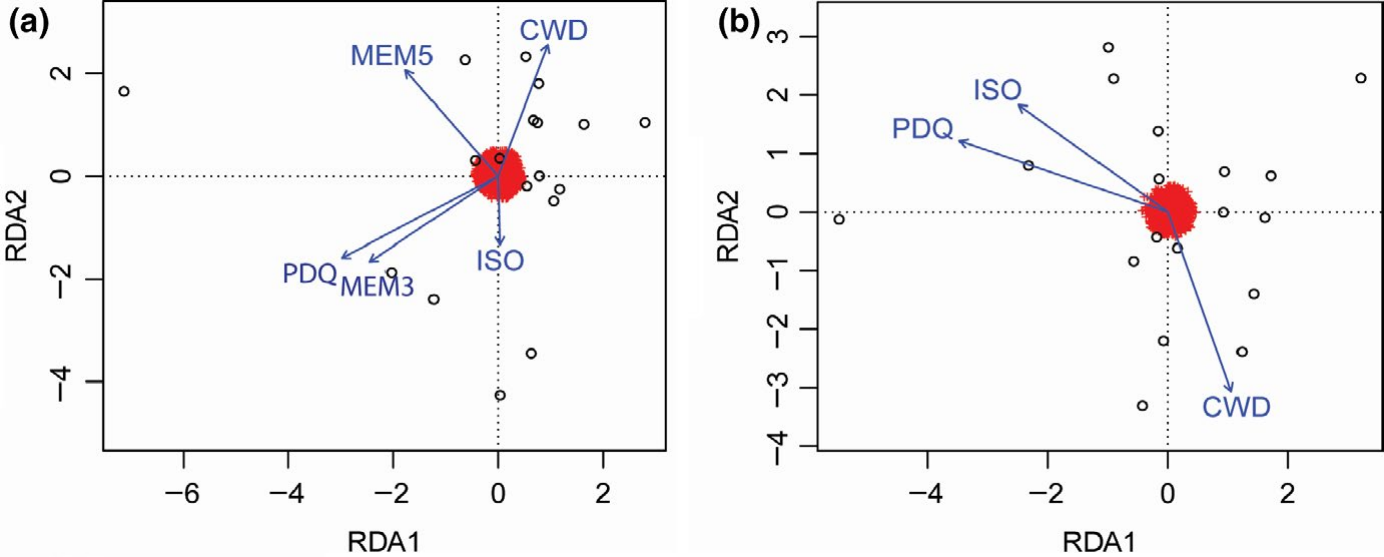
Triplot from Redundancy analysis showing how each explanatory variable affects the RDA axis with (a) representing the full RDA model and (b) a partial RDA model with the effects of climate conditioned on geography

We used the partial RDA model to detect outlier loci as candidates of importance in selection in a multivariate context, where allele frequencies were associated with climate after removing the effect of spatial predictors. Using the SNP loadings on the first RDA axis, we identified 24 outlier loci beyond the 95% confidence that demonstrated strong correlations to environmental variation, all of which were most correlated with precipitation of the driest quarter (PDQ) (Figure 5; Appendices S6 and S8). Our annotation procedure supported functional importance for seven of the 24 loci (Table 2).

### 3.4 Concordance among tests for signatures of selection

Overall, eight loci were detected as outliers by both RDA and LFMM. All of these loci were most associated with precipitation‐related variables (PC2 in LFMM and PDQ in RDA). Annotation through BLAST identified two of these loci as having a putative function (Table 2). Overlap was found between a BayeScan (*F*_ST_ outlier) and LFMM at one locus (1,123; Table 2).

### 3.5 Partitioning variation between climate and geographic space

The full RDA model explained 45% of the total variation in allele frequency and supported an influence of climate and/or space in shaping allelic variation (*p* = .001; adjusted *R* ^2^ = .22). The first two canonical axes from the full RDA model were significant (*p* = .002, and 0.013 respectively) and together accounted for 77% of the explained variation (Figure 5). The partial RDA model, with climate conditioned on space, was significant (*p* = .019; adjusted *R* ^2^ = .09) and constrained 49% of the variance explained by the full model. The first partial RDA axis was significant at the 0.1 level (*p* = .098) and accounted for 45% of the variation. The partial RDA model with space conditioned on climate accounted for 24% of the explained variation and was nonsignificant (*p* = .207). The remaining 27% of the explained variation was confounded between climate and geography.

## 4 DISCUSSION

Giant sequoia is a paleoendemic of California that has likely suffered from a long‐term demographic decline (Dodd & DeSilva, 2016). Today, it is limited to a number of restricted groves in the Sierra Nevada mountain chain. Small grove sizes and limited gene exchange among populations (DeSilva & Dodd, 2020) might be expected to limit its adaptive potential through inbreeding effects and genetic drift. However, through different approaches we have found evidence for a signal of spatially varying local adaptation associated with climate variables and, in particular, along gradients dominated by precipitation. We report here that population genetic structure in giant sequoia has been shaped by local adaptation overlain on historical population processes. From our study of genomic variation, we detected 79 loci as either *F*_ST_ outliers or loci with strong associations to climate as candidate regions of adaptive importance. Of these, we highlight 26 SNPs, found from multiple methods, or that correspond with functional annotation, as prime candidates for additional research. We emphasize that these outlier loci may include false positives and that experimental studies are needed to demonstrate functional significance of putative adaptive genomic regions (Barrett & Hoekstra, 2011; Kawecki & Ebert, 2004). Here, we present a first step toward understanding local adaptation in an iconic forest tree.

### 4.1 Population divergence and structure

We found evidence for strong population differentiation in our genomic data (*F*_ST_ = 0.15), which was close to our earlier estimate of *F*_ST_ = 0.14 from microsatellite variation (DeSilva & Dodd, 2020). Such high levels of differentiation are unusual in wind‐pollinated tree species, for which population differentiation is typically low and suggests that at least some populations have been isolated for a considerable time (McKay & Latta, 2002; Petit & Hampe, 2006). The 18 giant sequoia populations that we sampled, covering the range of the species, could be partitioned into nine clusters. Six of these clusters were restricted to northern isolated groves and the remaining three clusters include all southern populations that are somewhat more contiguous. Our results confirm the previously report of strong population structure among populations north of GRNT based on microsatellite data (DeSilva & Dodd, 2020; Dodd & DeSilva, 2016). Genetic diversity (unbiased heterozygosity) was no lower in the northern fragmented groves than in most populations within the range, supporting our earlier inference that northern groves have a long evolutionary history (Dodd & DeSilva, 2016). Moreover, estimates of uHe (calculated after removing putative adaptive sites; see Appendix S9) show consistent patterns across populations as previous estimates of He from microsatellite markers (DeSilva & Dodd, 2020), although uHe is lower in the SNP dataset. This pattern of population and genetic structure is unusual for north temperate conifers, for which higher latitude populations are commonly thought of as “leading edge” colonization following glacial retreat. Current groves extend above the lower extent of late Pleistocene glaciers (Moore and Moring 2013), so either some short distance upward colonization must have occurred, or pockets of unglaciated terrain may have served as very local refugia. Given the pattern of genomic diversity that we have detected and the long generation time (~300 years) of giant sequoia, it seems most likely that extant groves have either persisted through many generations or were colonized by short distance migrations.

### 4.2 Evidence for local adaptation

Despite the strong structure among populations, analysis of our genomic data revealed a signal of divergent selection associated with climatic variables. BayeScan detected few *F*_ST_ outliers due, in part, to the high population structure and increasing the prior odds for the neutral model to 100. However, each of these outliers exhibited high levels of differentiation (*F*_ST_ 0.55–0.72; Appendix S6). Univariate (LFMM) and multivariate (RDA) environmental association approaches identified more loci indicative of local adaptation. Although *F*_ST_ outlier approaches have been found to be more robust with respect to false positives than other methods (Lotterhos & Whitlock, 2014), environmental association studies are more successful in detecting loci under selection and can provide context for selective forces as well (De Mita et al. 2013). Our environmental association studies found climate to be an important predictor of allele frequency and accounted for the largest portion of explained variation after correcting for geographic space, a pattern consistent with local adaptation. Signatures of local adaptation to climate are prevalent in many tree species, including *Picea mariana*, *Alnus glutinosa*, *Populus trichocarpa*, *Cornus florida*, and *Quercus lobata* (De Kort et al., 2014; Geraldes et al., 2014; Pais et al., 2016; Prunier, Laroche, Beaulieu, & Bousquet, 2011; Sork et al., 2016). Moreover, the association of genomic variation with climate in giant sequoia is consistent with our previous work that found precipitation‐related variables play a role in patterns of isolation by environment at neutral genetic markers (DeSilva & Dodd, 2020). Here, a genome‐wide dataset that includes putative functional regions showed a signal of climatic factors shaping genomic variation, which suggests that local adaptation in situ, likely under conditions of limited gene flow, is important in this species.

Local adaptation is further supported by nine loci detected by multiple methods and 19 candidate loci with functional annotation (Table 2). Overlap in the detection of outlier loci has been reported in numerous field studies (Harrisson et al., 2017; Hess, Zendt, Matala, & Narum, 2016; Sork et al., 2016). A carefully designed simulation study demonstrated that overlap between GEA methods was found more often for actual targets of selection rather than false positives (Forester et al., 2018). In addition, two of the eight loci detected by both RDA and LFMM have relevant functional annotation, (loci 827 and 1,229; Table 2). A BLAST search suggests that Locus 827 is a kinesin‐like protein, KIN‐13A, which has been found to be involved in trichome morphogenesis (Lu, Lee, Pan, Maloof, & Liu, 2005). In plants, trichome occurrence and density are associated with increased drought resistance (Galdon‐Armero et al., 2018; Sletvold & Ågren, 2012). Locus 1,229 represents a potential pollen allergen gene, which is thought to be involved in plant responses to stress (Chen et al., 2016). Although our BLAST search suggested functional importance for 20 loci, many of the annotated regions are characterized only as mRNA with further functional roles yet to be determined (Table 2). The non‐annotated outlier loci are promising candidates for future research as they may be of unknown importance, linked to adaptive genes, in regulatory regions, or represent false positives. Any future annotation of the giant sequoia genome will provide valuable clarity as to the specific role of all outlier loci. Yet, we emphasize the candidate loci noted in this study demonstrate only strong associations with climate and identifying the exact targets of selection involves rigorous experimental research. Taken together, outlier loci with functional annotation or those detected by multiple methods provide strong support for adaptive variation across the range of giant sequoia.

### 4.3 Outlier SNPs driven by precipitation

Interestingly, variables associated with precipitation appeared to be the major drivers of local adaptation, which perhaps reflects the strong gradients of water relations on the western slope of the Sierra Nevada. Using gene–environment association methods (LFMM and RDA), we found evidence for adaptive differentiation across giant sequoia populations in response to gradients in precipitation and a more limited signal of local adaptation to temperature. LFMM analyses demonstrated seven times as many outliers correlated with precipitation‐related PC2 than to temperature‐related PC1 (Figure 4, Appendix S6). Although all RDA outliers were correlated with three environmental factors, PDQ, a measure of summer precipitation, CWD, a measure of aridity, and ISO, a measure diurnal and annual temperature fluctuation, outlier loci showed the strongest relationship to PDQ (Appendix S8). Thus, both LFMM and RDA indicate a subtle signal of adaptation to temperature and a stronger signature of divergent selection in response to gradients in water‐related variables. Gradients of water availability are important selective agents for many tree species including *Picea mariana*, *Cornus florida*, *Fagus sylvatica*, *Quercus* spp., and *Pinus albicaulis* (Lind et al., 2017; Martins et al., 2018; Pais et al., 2016; Pluess et al., 2016; Prunier et al., 2011; Sork et al., 2016). In addition, many ecological studies have noted that giant sequoia is sensitive to water availability during its establishment phase (Hartesveldt, Harvey, Shellhammer, & Stecker, 1975; Rundel, 1972; Shellhammer & Shellhammer, 2006; York, Battles, & Heald, 2003). Giant sequoia is known to have bursts of reproduction after fire and subsequently experience high seedling mortality due to desiccation (Weatherspoon, 1990). Considering the reproductive biology of this species, water availability is a highly plausible selective agent. For Mediterranean type climates, PDQ is of particular relevance for desiccation sensitive species as it equates to summer precipitation, which may represent a vital water source for giant sequoia seedlings during a vulnerable establishment phase.

### 4.4 Limitations, opportunities, and future implications

It is important to note that GEA methods can suffer from low power or high false‐positive rate under some demographic scenarios. Although LFMM has been shown to be robust to various demographic scenarios, including those that create high levels of population structure, this method can have elevated false discovery rates (FDR) under scenarios that create IBD (Forester, Jones, Joost, Landguth, & Lasky, 2016; Lotterhos & Whitlock, 2015; de Villemereuil et al., 2014). Here, we do not find a significant signal of IBD in our data. Yet, the role of IBD or other neutral factors affecting population structure in giant sequoia has not been fully elucidated. Previous research has indicated isolation by distance (IBD) and/or ancient divergence separating the northern and southern populations of giant sequoia (DeSilva & Dodd, 2020; Dodd & DeSilva, 2016). Here, a large portion of the explained variance in our data (27%) was confounded between climatic variation and geographic space, which is perhaps due, to the strictly north‐south range of giant sequoia, making it inherently difficult to decouple distance from environmental gradients that vary latitudinally. Therefore, LFMM results should be treated with some caution due to the potential contribution of IBD to population structure. In contrast to LFMM, RDA models show high power and low false‐positive rates under IBD (Forester et al., 2016). Simulation studies indicate that the performance of RDA also remains high when population structure and selective gradients are explicitly correlated (Capblancq et al., 2018). Yet, RDA is not without limitations, as it can have low power under island demographic models (Forester et al., 2018) or when selective pressures are highly clustered (Capblancq et al., 2018). Given that each GEA method has particular limitations, we believe the outlier loci detected by both LFMM and RDA, as well as outliers with functional annotation remain strong candidate regions of adaptive importance.

There is an ongoing need for future studies to provide additional clarity on the distribution of adaptive variation and genetic architecture of local adaptation in giant sequoia. To our knowledge, this study represents the first investigation of adaptive variation using genome‐wide data. Yet, the results presented here are based on a small subset of the genomic variation within the species, as the giant sequoia genome is very large (8.5 Gb, Redwood Genome Project). More comprehensive sampling of the genome as well as an incorporation of phenotypic information will greatly improve our understanding of local adaptation in this species. In addition, future annotation of the giant sequoia genome will provide opportunities to better understand the genetic underpinnings of many phenotypic traits.

A trend toward increased aridity along midelevation Sierra Nevada forests could undermine the long‐term persistence of giant sequoia. With end‐of‐century predictions for this region that include decreasing snowfall and earlier snowmelt, forests of the Sierra Nevada mountains will likely experience an accentuation of the summer drought that is typical of Mediterranean climates (Fyfe et al., 2017; Stewart, Cayan, & Dettinger, 2004; Sun et al., 2018). Considering the evidence presented here, we highlight the potential that increased water stress may create maladaptation of giant sequoia populations to their environment. It has been suggested that a long‐term decline (over the last ~2My) of giant sequoia is tied to increasing aridity during the development of current climate regimes (Dodd & DeSilva, 2016). Moreover, some giant sequoia populations suffered extensive foliage die back during the drought period from 2012 to 2016 (Stephenson et al., 2018), providing further indication of sensitivity of giant sequoia to arid conditions.

## 5 CONCLUSIONS

We provide evidence of local adaptation along gradients of precipitation and highlight genomic regions of potential adaptive importance for additional research. This information can aid in determining the best course of action to preserve giant sequoia into the future. Locally adapted populations of giant sequoia are facing an accentuation of summer drought to which they may be maladapted. Genomic variation currently present in more arid regions of the giant sequoia range could include “preadapted” variants that might enhance the adaptive response of nearby populations (Aitken & Bemmels, 2016; Kremer et al., 2012). Currently, DCRK, GRNT, MCTR, and RMNT inhabit areas experiencing the lowest levels of summer precipitation (PDQ) and thus may provide potential sources of adaptive alleles. Given the limited gene flow in much of giant sequoia range, it is unlikely this variation will spread quickly by natural means (DeSilva & Dodd, 2020). Thus, forest managers may consider assisting in the movement of genetic resources in order to enhance the adaptive potential of giant sequoia populations.

## CONFLICT OF INTEREST

The authors declare no conflicts of interest.

## AUTHOR CONTRIBUTION

**Rainbow DeSilva:** Conceptualization (lead); Data curation (lead); Formal analysis (lead); Methodology (lead); Writing‐original draft (lead); Writing‐review & editing (equal). **Richard S. Dodd:** Conceptualization (supporting); Formal analysis (supporting); Funding acquisition (lead); Methodology (supporting); Supervision (lead); Writing‐original draft (supporting); Writing‐review & editing (equal).

## Supporting information

Appendix S1

Appendix S2

Appendix S3

Appendix S4

Appendix S5

Appendix S6

Appendix S7

Appendix S8

Appendix S9

## Data Availability

The full SNP data are available through DRYAD https://doi.org/10.6078/D1GT4D
