# Supplementary material for: Association of genetic and climatic variability in giant sequoia, Sequoiadendron giganteum, reveals signatures of local adaptation along moisture‐related gradients
Source: Ecol Evol. 2020 Sep 1;10(19):10619–32. doi: 10.1002/ece3.6716 (PMC7548164; doi:10.1002/ece3.6716)
Supplement: Supplementary file 1 — Appendix S1 [file ECE3-10-10619-s001.docx]

| **Filtering Step** | **Number of Contigs** | **Number of SNPs** |
| --- | --- | --- |
| Total Variable sites | 8701 | 3,915,935 |
| Removal of loci with uneven mapping quality | 8333 | 1,344,117 |
| Removal of unbalanced reads in heterozygotes | 8329 | 1,300,206 |
| Remove sites with average read depth >200 | 8329 | 1,299,461 |
| Remove samples with read depth <5 | 8329 | 1,299,461 |
| Remove sites with MAC <3 | 7557 | 244,489 |
| Remove sites with >80% missing data | 1133 | 4,276 |
| Remove Indels | 1120 | 4,235 |
| Remove multiallelic sites | 1118 | 4,208 |
| Thinning to 1-SNP per fragment | 1118 | 1,364 |

Appendix S1: Additional data filtering details: Number of Contigs and SNP’s at each filtering step.
