# Supplementary material for: Association of genetic and climatic variability in giant sequoia, Sequoiadendron giganteum, reveals signatures of local adaptation along moisture‐related gradients
Source: Ecol Evol. 2020 Sep 1;10(19):10619–32. doi: 10.1002/ece3.6716 (PMC7548164; doi:10.1002/ece3.6716)
Supplement: Supplementary file 2 — Appendix S2 [file ECE3-10-10619-s002.docx]

**Appendix S2:** Variable loadings and proportion of variance explained by each axis for Principal Components Analysis

| Environmental Variable | Loadings on PC1 (47% of variance) | Loadings on PC2 (35% of variance) |
| --- | --- | --- |
| Annual Mean Temperature | 0.30533764 | 0.08895816 |
| Mean Diurnal Range | 0.23382154 | -0.1319765 |
| Isothermality | 0.14402502 | -0.1584969 |
| Temperature Seasonality | 0.2610051 | -0.0711812 |
| May Temperature of Warmest Month | 0.3128064 | 0.00651353 |
| Min Temperature of Coldest Month | 0.25664444 | 0.15588655 |
| Temperature Annual Range | 0.26828944 | -0.0980023 |
| Mean Temperature of Wettest Quarter | 0.27880069 | 0.16738419 |
| Mean Temperature of Driest Quarter | 0.30597282 | 0.07812717 |
| Mean Temperature of Warmest Quarter | 0.30670671 | 0.07428924 |
| Mean Temperature of Coldest Quarter | 0.28724379 | 0.1406514 |
| Annual Precipitation | 0.12018293 | -0.3250514 |
| Precipitation of Wettest Month | 0.11053911 | -0.3215777 |
| Precipitation of Driest Month | -0.1120967 | -0.3191191 |
| Precipitation Seasonality | 0.14263436 | 0.12207502 |
| Precipitation of Wettest Quarter | 0.11777354 | -0.3202812 |
| Precipitation of Driest Quarter | 0.04615045 | -0.3459344 |
| Precipitation of Warmest Quarter | -0.0576154 | -0.3355527 |
| Precipitation of Coldest Quarter | 0.11802076 | -0.3218542 |
| Elevation | -0.2902339 | 0.08573527 |
| Climate Water Deficit | 0.05050955 | 0.28198092 |
