# Supplementary material for: Association of genetic and climatic variability in giant sequoia, Sequoiadendron giganteum, reveals signatures of local adaptation along moisture‐related gradients
Source: Ecol Evol. 2020 Sep 1;10(19):10619–32. doi: 10.1002/ece3.6716 (PMC7548164; doi:10.1002/ece3.6716)
Supplement: Supplementary file 3 — Appendix S3 [file ECE3-10-10619-s003.docx]

**Appendix S3:** PCA plot showing the behavior of the climate variables across PC1 and PC2. Genomic data is represented by individual ID’s* and blue shading represents populations, with color becoming lighter from North-to-South.

*Individual ID’s have been shortened to two letter codes to improve figure clarity
