# Supplementary material for: Association of genetic and climatic variability in giant sequoia, Sequoiadendron giganteum, reveals signatures of local adaptation along moisture‐related gradients
Source: Ecol Evol. 2020 Sep 1;10(19):10619–32. doi: 10.1002/ece3.6716 (PMC7548164; doi:10.1002/ece3.6716)
Supplement: Supplementary file 4 — Appendix S4 [file ECE3-10-10619-s004.docx]

Appendix S4: Full AMOVA results

| Source of Variation | F-stat | F-value | Std.Dev. | c.i.2.5% | c.i.97.5% | P-value |
| --- | --- | --- | --- | --- | --- | --- |
| Among population | F_st | 0.151 | 0.005 | 0.141 | 0.161 | 0.000 |
| Among Group | F_ct | 0.023 | 0.002 | 0.018 | 0.027 | 0.000 |
