# Supplementary material for: Association of genetic and climatic variability in giant sequoia, Sequoiadendron giganteum, reveals signatures of local adaptation along moisture‐related gradients
Source: Ecol Evol. 2020 Sep 1;10(19):10619–32. doi: 10.1002/ece3.6716 (PMC7548164; doi:10.1002/ece3.6716)
Supplement: Supplementary file 5 — Appendix S5 [file ECE3-10-10619-s005.docx]

**Appendix S5:** Details from STRUCTURE analysis: Likelihood and standard deviation for K 7-11.

| Number of Clusters | Mean Likelihood | SD |
| --- | --- | --- |
| K = 7 | -68470.675 | 322.230 |
| K = 8 | -68664.85 | 1426.305 |
| K = 9 | -67287.45 | 130.523 |
| K = 10 | -67319.475 | 810.725 |
| K = 11 | -66549.65 | 96.078 |
