# Supplementary material for: Association of genetic and climatic variability in giant sequoia, Sequoiadendron giganteum, reveals signatures of local adaptation along moisture‐related gradients
Source: Ecol Evol. 2020 Sep 1;10(19):10619–32. doi: 10.1002/ece3.6716 (PMC7548164; doi:10.1002/ece3.6716)
Supplement: Supplementary file 6 — Appendix S6 [file ECE3-10-10619-s006.docx]

Appendix S6: All outlier loci detected by BayeScan, LFMM, and RDA

| Locus ID | Detection Method | Associated Variable | F_ST_ | Adjusted P-value | Axis 1 Loading | Annotation |
| --- | --- | --- | --- | --- | --- | --- |
| 522 | BS | -- | 0.59 | -- | -- | -- |
| 701 | BS | -- | 0.72 | -- | -- | -- |
| 801 | BS | -- | 0.55 | -- | -- | -- |
| 828 | BS | -- | 0.58 | -- | -- | Unknown mRNA |
| 1324 | BS | -- | 0.55 | -- | -- | -- |
| 1123 | BS, LFMM | PC1 | 0.66 | 1.72E-04 | -- | -- |
| 186 | LFMM | PC1 |  | 7.39E-06 | -- | Unknown mRNA |
| 218 | LFMM | PC1 |  | 5.50E-05 | -- | Unknown mRNA |
| 385 | LFMM | PC1 |  | 3.07E-07 | -- | -- |
| 452 | LFMM | PC1 |  | 5.30E-05 | -- | Unknown mRNA |
| 470 | LFMM | PC1 |  | 1.39E-04 | -- | -- |
| 604 | LFMM | PC1 |  | 9.89E-05 | -- | NA |
| 92 | LFMM | PC2 |  | 2.99E-05 | -- | -- |
| 125 | LFMM | PC2 |  | 8.62E-04 | -- | -- |
| 126 | LFMM | PC2 |  | 1.59E-03 | -- | -- |
| 151 | LFMM | PC2 |  | 5.59E-05 | -- | -- |
| 193 | LFMM | PC2 |  | 7.66E-06 | -- | -- |
| 196 | LFMM | PC2 |  | 9.55E-04 | -- | -- |
| 251 | LFMM | PC2 |  | 1.91E-05 | -- | Unknown mRNA |
| 253 | LFMM | PC2 |  | 4.10E-04 | -- | -- |
| 306 | LFMM | PC2 |  | 2.14E-04 | -- | -- |
| 319 | LFMM | PC2 |  | 1.78E-03 | -- | -- |
| 351 | LFMM | PC2 |  | 1.22E-03 | -- | -- |
| 518 | LFMM | PC2 |  | 8.94E-05 | -- | -- |
| 526 | LFMM | PC2 |  | 1.05E-03 | -- | -- |
| 547 | LFMM | PC2 |  | 5.54E-04 | -- | -- |
| 552 | LFMM | PC2 |  | 4.40E-05 | -- | -- |
| 562 | LFMM | PC2 |  | 1.06E-03 | -- | -- |
| 641 | LFMM | PC2 |  | 9.30E-04 | -- | -- |
| 653 | LFMM | PC2 |  | 7.57E-04 | -- | -- |
| 654 | LFMM | PC2 |  | 7.42E-04 | -- | -- |
| 690 | LFMM | PC2 |  | 3.01E-05 | -- | -- |
| 693 | LFMM | PC2 |  | 7.09E-04 | -- | -- |
| 695 | LFMM | PC2 |  | 1.24E-03 | -- | -- |
| 722 | LFMM | PC2 |  | 1.52E-03 | -- | Unknown mRNA |
| 730 | LFMM | PC2 |  | 8.95E-05 | -- | -- |
| 764 | LFMM | PC2 |  | 5.68E-04 | -- | -- |
| 868 | LFMM | PC2 |  | 1.07E-03 | -- | -- |
| 870 | LFMM | PC2 |  | 9.48E-04 | -- | Unknown mRNA |
| 935 | LFMM | PC2 |  | 6.01E-05 | -- | -- |
| 1024 | LFMM | PC2 |  | 7.43E-04 | -- | Retrotransposon |
| 1029 | LFMM | PC2 |  | 6.56E-06 | -- | Unknown mRNA |
| 1054 | LFMM | PC2 |  | 1.04E-03 | -- | -- |
| 1059 | LFMM | PC2 |  | 1.04E-03 | -- | -- |
| 1062 | LFMM | PC2 |  | 1.66E-04 | -- | Unknown mRNA |
| 1076 | LFMM | PC2 |  | 8.18E-04 | -- | -- |
| 1115 | LFMM | PC2 |  | 1.41E-05 | -- | -- |
| 1167 | LFMM | PC2 |  | 9.34E-05 | -- | -- |
| 1191 | LFMM | PC2 |  | 2.34E-05 | -- | -- |
| 1214 | LFMM | PC2 |  | 1.52E-03 | -- | magnesium transporter MRS2-4-like |
| 1253 | LFMM | PC2 |  | 2.91E-04 | -- | Unknown mRNA |
| 1295 | LFMM | PC2 |  | 1.43E-03 | -- | -- |
| 1307 | LFMM | PC2 |  | 1.28E-03 | -- | -- |
| 1313 | LFMM | PC2 |  | 5.87E-06 | -- | pleiotropic drug resistance protein 1-like |
| 1332 | LFMM | PC2 |  | 1.46E-03 | -- | -- |
| 368 | LFMM, RDA | PC2, PDQ |  | 2.35E-05 | -0.211321 | -- |
| 421 | LFMM, RDA | PC2, PDQ |  | 9.56E-05 | -0.194232 | -- |
| 471 | LFMM, RDA | PC2, PDQ |  | 2.03E-05 | -0.198918 | -- |
| 515 | LFMM, RDA | PC2, PDQ |  | 1.46E-03 | -0.211814 | -- |
| 679 | LFMM, RDA | PC2, PDQ |  | 3.51E-04 | -0.223479 | -- |
| 827 | LFMM, RDA | PC2, PDQ |  | 9.12E-04 | -0.205785 | kinesin-like protein KIN-13A mRNA |
| 1229 | LFMM, RDA | PC2, PDQ |  | 1.65E-03 | -0.178222 | pollen allergen gene |
| 1286 | LFMM, RDA | PC2, PDQ |  | 4.41E-07 | 0.263690 | -- |
| 5 | RDA | PDQ |  | -- | -0.171565 | -- |
| 90 | RDA | PDQ |  | -- | -0.167273 | -- |
| 122 | RDA | PDQ |  | -- | -0.167507 | - |
| 166 | RDA | PDQ |  | -- | -0.202460 | -- |
| 273 | RDA | PDQ |  | -- | -0.166244 | -- |
| 338 | RDA | PDQ |  | -- | -0.187693 | Wall-associated receptor kinase-like 1 |
| 494 | RDA | PDQ |  | -- | -0.163779 | -- |
| 572 | RDA | PDQ |  | -- | -0.171818 | -- |
| 612 | RDA | PDQ |  | -- | -0.242672 | arogenate dehydratase gene |
| 617 | RDA | PDQ |  | -- | -0.223479 | Unknown mRNA |
| 673 | RDA | PDQ |  | -- | -0.175162 | -- |
| 709 | RDA | PDQ |  | -- | -0.175226 | -- |
| 940 | RDA | PDQ |  | -- | 0.235355 | signal peptidase I AT2G30440 mRNA |
| 1066 | RDA | PDQ |  | -- | -0.195195 | Unknown mRNA |
| 1116 | RDA | PDQ |  | -- | -0.163279 | -- |
| 1305 | RDA | PDQ |  | -- | 0.235376 | -- |
