# Supplementary material for: Association of genetic and climatic variability in giant sequoia, Sequoiadendron giganteum, reveals signatures of local adaptation along moisture‐related gradients
Source: Ecol Evol. 2020 Sep 1;10(19):10619–32. doi: 10.1002/ece3.6716 (PMC7548164; doi:10.1002/ece3.6716)
Supplement: Supplementary file 7 — Appendix S7 [file ECE3-10-10619-s007.docx]

**Appendix S7**: Histograms of adjusted p-values for (LFMM), with the number of latent factors (K) set from 8-12.
