# Supplementary material for: Association of genetic and climatic variability in giant sequoia, Sequoiadendron giganteum, reveals signatures of local adaptation along moisture‐related gradients
Source: Ecol Evol. 2020 Sep 1;10(19):10619–32. doi: 10.1002/ece3.6716 (PMC7548164; doi:10.1002/ece3.6716)
Supplement: Supplementary file 8 — Appendix S8 [file ECE3-10-10619-s008.docx]

**Appendix S8:** Results from partial redundancy analysis, with climate conditioned on geographic space, showing loadings of climate variables on RDA axis 1, and the relative contribution of each climate factor.

| **SNP_ID** | **Loading** | **PDQ** | **ISO** | **CWD** |
| --- | --- | --- | --- | --- |
| 5 | -0.172 | 0.585 | 0.237 | -0.079 |
| 90 | -0.167 | 0.529 | 0.028 | -0.024 |
| 122 | -0.168 | 0.533 | 0.072 | -0.169 |
| 166 | -0.202 | 0.748 | 0.165 | -0.313 |
| 273 | -0.166 | 0.294 | 0.046 | 0.266 |
| 338 | -0.188 | 0.584 | 0.258 | -0.189 |
| 368 | -0.211 | 0.607 | 0.321 | -0.084 |
| 421 | -0.194 | 0.822 | 0.257 | -0.599 |
| 471 | -0.199 | 0.633 | 0.163 | -0.144 |
| 494 | -0.164 | 0.768 | 0.294 | -0.584 |
| 515 | -0.212 | 0.696 | 0.265 | -0.229 |
| 572 | -0.172 | 0.470 | -0.089 | 0.040 |
| 612 | -0.243 | 0.690 | 0.252 | -0.208 |
| 617 | -0.171 | 0.713 | 0.278 | -0.351 |
| 673 | -0.175 | 0.603 | 0.158 | -0.161 |
| 679 | -0.223 | 0.710 | 0.635 | -0.397 |
| 709 | -0.175 | 0.619 | -0.069 | -0.194 |
| 827 | -0.206 | 0.682 | 0.012 | -0.148 |
| 940 | 0.235 | -0.755 | -0.048 | 0.300 |
| 1066 | -0.195 | 0.632 | 0.258 | -0.257 |
| 1116 | -0.163 | 0.590 | 0.212 | -0.205 |
| 1229 | -0.178 | 0.429 | 0.219 | -0.115 |
| 1286 | 0.264 | -0.812 | -0.294 | 0.389 |
| 1305 | 0.235 | -0.710 | -0.364 | 0.183 |
