# Supplementary material for: Association of genetic and climatic variability in giant sequoia, Sequoiadendron giganteum, reveals signatures of local adaptation along moisture‐related gradients
Source: Ecol Evol. 2020 Sep 1;10(19):10619–32. doi: 10.1002/ece3.6716 (PMC7548164; doi:10.1002/ece3.6716)
Supplement: Supplementary file 9 — Appendix S9 [file ECE3-10-10619-s009.docx]

**Appendix S9:** Population information and Genetic diversity summary statistics calculated for each population after removal of putative adaptive loci

| Grove Name | Population Code | GPS Location | Sample Size | Ho | uHe | F_IS_ | G’st (average) |
| --- | --- | --- | --- | --- | --- | --- | --- |
| Placer | PLAC | 39.06, -120.57 | 6 | 0.09 | 0.07 | 0.02 | 0.34 |
| North Calaveras | CALN | 38.28, -120.30 | 8 | 0.14 | 0.18 | 0.16 | 0.17 |
| South Calaveras | CALS | 38.24, -120.25 | 8 | 0.15 | 0.18 | 0.13 | 0.15 |
| Tuolumne | TUOL | 37.77, -119.81 | 8 | 0.16 | 0.16 | -0.03 | 0.25 |
| Merced | MERC | 37.75, -119.84 | 8 | 0.16 | 0.17 | 0.00 | 0.21 |
| Mariposa | MPSA | 37.51, -119.60 | 8 | 0.13 | 0.14 | 0.12 | 0.20 |
| Nelder | NELD | 37.43, -119.59 | 8 | 0.16 | 0.19 | 0.12 | 0.14 |
| McKinley | MKLY | 37.03, -119.11 | 8 | 0.15 | 0.17 | 0.07 | 0.17 |
| Grant | GRNT | 36.75, -118.97 | 8 | 0.12 | 0.13 | 0.17 | 0.15 |
| Redwood Mountain | RMNT | 36.60, -118.92 | 8 | 0.12 | 0.11 | 0.16 | 0.13 |
| Giant Forest | GFOR | 36.57, -118.76 | 8 | 0.15 | 0.20 | 0.18 | 0.09 |
| Atwell | ATWL | 36.47, -118.67 | 8 | 0.17 | 0.21 | 0.08 | 0.11 |
| Mcintyre | MCTR | 36.13, -118.58 | 8 | 0.13 | 0.11 | 0.09 | 0.13 |
| Freeman Creek | FMAN | 36.14, -118.52 | 8 | 0.17 | 0.18 | 0.08 | 0.11 |
| Long Meadow | LMDW | 35.96, -118.60 | 8 | 0.12 | 0.11 | 0.16 | 0.16 |
| Cunningham | CNHM | 35.92, -118.57 | 9 | 0.16 | 0.17 | 0.00 | 0.16 |
| Packsaddle | PKSD | 35.93, -118.59 | 8 | 0.15 | 0.15 | 0.08 | 0.16 |
| Deer Creek | DCRK | 35.88, -118.61 | 8 | 0.16 | 0.18 | 0.04 | 0.16 |
